# *Lactobacillus iners* Is Associated with Vaginal Dysbiosis in Healthy Pregnant Women: A Preliminary Study

**DOI:** 10.1155/2019/6079734

**Published:** 2019-10-23

**Authors:** Nengneng Zheng, Renyong Guo, Yinyu Yao, Meiyuan Jin, Yiwen Cheng, Zongxin Ling

**Affiliations:** ^1^Department of Obstetrics, Tongde Hospital of Zhejiang Province, Hangzhou, Zhejiang 310012, China; ^2^Department of Laboratory Medicine, First Affiliated Hospital, College of Medicine, Zhejiang University, Key Laboratory of Clinical In Vitro Diagnostic Techniques of Zhejiang Province, Hangzhou, Zhejiang 310003, China; ^3^Collaborative Innovation Center for Diagnosis and Treatment of Infectious Diseases, State Key Laboratory for Diagnosis and Treatment of Infectious Diseases, National Clinical Research Center for Infectious Diseases, The First Affiliated Hospital, School of Medicine, Zhejiang University, Hangzhou, Zhejiang 310003, China

## Abstract

Vaginal dysbiosis has been identified to be associated with adverse pregnancy outcomes, such as preterm delivery and premature rupture of membranes. However, the overall structure and composition of vaginal microbiota in different trimesters of the pregnant women has not been fully elucidated. In this study, the physiological changes of the vaginal microbiota in healthy pregnant women were investigated. A total of 83 healthy pregnant participants were enrolled, who are in the first, second, or third pregnancy trimester. Quantitative real-time PCR was used to explore the abundant bacteria in the vaginal microbiota. No significant difference in the abundance of *Gardnerella*, *Atopobium*, *Megasphaera*, *Eggerthella*, *Leptotrichia*/*Sneathia*, or *Prevotella* was found among different trimesters, except *Lactobacillus*. Compared with the first pregnancy trimester, the abundance of *L. iners* decreased in the second and third trimester while the abundance of *L. crispatus* was increased in the second trimester. Moreover, we also found that vaginal cleanliness is correlated with the present of *Lactobacillus*, *Atopobium*, and *Prevotella* and leukocyte esterase is associated with *Lactobacillus*, *Atopobium*, *Gardnerella*, *Eggerthella*, *Leptotrichia*/*Sneathia*, and *Prevotella*. For those whose vaginal cleanliness raised or leukocyte esterase became positive, the richness of *L. iners* increased, while that of *L. crispatus* decreased significantly. Our present data indicated that the altered vaginal microbiota, mainly *Lactobacillus*, could be observed among different trimesters of pregnancy and *L. iners* could be considered as a potential bacterial marker for evaluating vaginal cleanliness and leukocyte esterase.

## 1. Introduction

The vaginal microbiota consist of a variety of species, including both anaerobic and aerobic microorganisms [1]. These commensal microorganisms in the vagina provide beneficial effects against opportunistic and pathogenic bacteria, constituting the first line of defense against invasive microorganisms [2]. Some of the species such as *L. crispatus*, *L. gasseri*, and *L. jensenii* take function by lowering the pH and production of large amounts of lactic acid and bactericide compounds, such as hydrogen peroxide (H_2_O_2_) and bacteriocins [3, 4], which prevent possible pathogenic or opportunistic pathogenic bacteria and ensure vaginal epithelial homeostasis.

Preterm delivery is known to be one of the main causes of perinatal mortality and morbidity worldwide [5]. Generally, it will result in very low birth weight, prolonged stay in hospital, increased risk of chronic lung disease, and even cerebral palsy. Besides, premature rupture of membranes (PROM) is also a dangerous complication. In the event of PROM, the uterine cavity, placenta, and fetus are exposed to ascending infection and increased risk of chorioamnionitis and funisitis, which are both associated with poor maternal and neonatal outcomes [6–10]. Recently, lower genital tract infection has been recognized as one of the more important risk factors associated with preterm delivery and PROM, especially in healthy nulliparous women [11]. Dysbiosis of vaginal microbiome has been recognized as a potential cause of adverse pregnancy outcomes, if they are not dominated by *Lactobacillus* [12–15]. However, the influence of vaginal dysbiosis on preterm delivery and PROM still remains unknown.

Healthy pregnancy is characterized by a temporary dynamic shift towards stable, reduced richness and low diversity in the community structures dominated by *Lactobacillus* spp. which aids in the prevention of pathogenic bacteria, different from the vaginal microbiota composition of nonpregnant women [16, 17]. Bacterial vaginosis (BV) is a condition in which the vaginal microbiota suffer a reduction in several species of probiotic *Lactobacillus* and an increase in the presence of anaerobes (*Gardnerella vaginalis*, *Atopobium vaginae*, and *Mobiluncus* sp.), which has been shown in our previous studies [18, 19]. It is hypothesized that colonization of the pathogenic bacteria in the vagina activates the local and upper (cervical and fetal membrane) innate immune system, drives an inflammatory cascade, and leads to a remodeling and disruption of membrane architecture and preterm delivery or PROM [20, 21].

Currently, cultivation-independent molecular approaches are favored for overcoming the difficult cultivation conditions such as nutrition and anaerobic requirements [22]. Thus, they have been used to demonstrate greater vaginal microbial diversity than that recognized previously. Several cultivation-independent species, such as *L. iners* and *A. vaginae*, have been identified as important elements of the vaginal microbiota as they are found across *Lactobacillus*-dominated and non-*Lactobacillus*-dominated vaginal microbiota groups [23, 24]. Thus, a deeper understanding of the composition and structure of the vaginal microbiota in healthy pregnant women is essential for fully elucidating the etiology of vaginal diseases as well as for the prevention and treatment of such diseases. Furthermore, there is an urgent need for a better understanding of when the clinical relevance of some bacteria became increasingly apparent, due to a change in either relative or absolute abundance.

The aim of this study was to investigate the vaginal microbiota in the first, second, and third pregnancy trimester in healthy pregnant women using a cultivation-independent approach. The exploration of the vaginal microbiota in these women provides a theoretical basis that may help to prevent vaginal infections during pregnancy and reduce preterm delivery and PROM.

## 2. Materials and Methods

### 2.1. Subjects' Recruitment

Healthy pregnant women, without abnormal vaginal discharge, were randomly enrolled, when they came to the Department of Obstetrics of Tongde Hospital in Zhejiang Province (Zhejiang, China) for routine medical examination between July and November 2016. A total of 83 subjects, aged 30.0 ± 4.6 years, were studied across three time points, with 33 between 5 and 10 weeks' gestation in the first trimester, 24 between 20 and 27 weeks' gestation in the second trimester, and 26 between 37 and 39 weeks' gestation in the third trimester. The following criteria were used to exclude subjects: a history of preterm delivery; previous multiple pregnancies; placenta previa or vaginal bleeding; candidiasis, BV, or trichomoniasis; use of antibiotics, probiotics, prebiotics, or synbiotics in the previous month; cervical incompetence or suspected uterine malformation; vaginal intercourse within the last 3 days; or were immunocompromised. Informed written consent was obtained from each of the participants before enrollment. This research was conducted in agreement with the ethical principles of the Declaration of Helsinki, and the study protocol was approved by the Ethics Committee of the Tongde Hospital of Zhejiang Province.

### 2.2. Sample Collection

Three swabs of vaginal secretions were taken from the posterior wall of the vaginal fornix at an outpatient service when these participants underwent speculum examination. Two of the swabs were applied onto a slide for determination of vaginal pH and vaginal infection status. The remaining vaginal swab was covered, placed on ice, and used for bacterial genomic DNA extraction. Vaginal pH was measured using a pH strip (Sanaisi Company, Shanghai, China). Vaginal infection status was determined by examining a wet mount smear in potassium hydroxide for detection of candidiasis and in saline for detection of motile trichomonas and clue cells. BV diagnosis was assessed according to Amsel clinical criteria [25]. H_2_O_2_ and leukocyte esterase were analyzed according to the manual method of the BV union diagnosis kits (Lizhu Company, Zhuhai, Guangdong, China). Vaginal cleanliness was evaluated according to morphological observations ([Supplementary-material supplementary-material-1]) [26]. The criteria of vaginal cleanliness were as follows [27]: Grade I was a large number of large Gram-positive rods (indicative of *Lactobacillus* spp.), vaginal epithelial cells, and no other bacteria observed with WBC 0–5/HP under microscopy. Grade II was some *Lactobacillus* spp. and vaginal epithelial cells, some pus cells, and other bacteria observed under microscopy with WBC 10–15/HP. Grade III was a small amount of *Lactobacillus* spp., a large number of pus cells, and other bacteria observed under microscopy with WBC 15–30/HP. Grade IV was no *Lactobacillus* spp. but pus cells and other bacteria observed under microscopy with WBC more than 30/HP. Grades I-II mean normal vaginal cleanliness, while grades III-IV mean abnormal vaginal cleanliness with inflammation. The vaginal swabs taken for bacterial genomic DNA extraction were transferred to the laboratory immediately and stored at −80°C until analyzed. Additionally, hospital records were reviewed following delivery to determine pregnancy outcome, including preterm birth, premature rupture of membranes, or preterm premature rupture of membranes.

### 2.3. Total Bacterial Genomic DNA Extraction

The bacterial cells retrieved on swabs were submerged in 1 mL of ice-cold phosphate-buffered saline (pH 7.5) and vigorously agitated to dislodge cells. The suspension was then transferred to a tube containing 100 mg of glass beads (Promega Corporation, Madison, WI, USA) and placed into a FastPrep FP120 instrument (Thermo Scientific, Pittsburgh, PA, USA) to disrupt the cell membranes. The suspension was then centrifuged at 17,000 g for 10 min, resuspended in 400 *μ*L of lysozyme, and incubated for 1 h at 37°C. Subsequently, 500 *μ*L of cell lysis solution was added and warmed for an additional 30 min at 37°C. The reaction solution was then centrifuged for 10 min at 12,000 g. After removing the supernatant, 180 *μ*L of lysis buffer (buffer ATL) and 25 *μ*L of proteinase K were added to the sample and incubated for 60 min at 55°C. The QIAamp DNA Mini Extraction Kit (QIAGEN, Hilden, Germany) was then used for the isolation of the genomic DNA according to the manufacturer's instructions. One sterile cotton swab was used as a negative control alongside the patient specimens. The concentration of extracted DNA was determined using a Smart-Spec Plus spectrophotometer (Bio-Rad Laboratories, Hercules, CA, USA). Its integrity and size were checked by 1.0% agarose gel electrophoresis, containing 0.5 mg/ml ethidium bromide. All DNA was stored at −20°C before analysis.

### 2.4. Quantitative Real-Time PCR (qPCR) Analysis

The ABI 7900 Fast Real-Time PCR instrument and Sequence Detection Software version 1.6.3 (Applied Biosystems, Foster City, CA, USA) were used to perform qPCR on all subjects, to determine accurate copy numbers of bacteria in the vaginal specimens. The following species-specific primer sets were chosen to quantify total bacteria in the samples, *Lactobacillus* genus, *L. crispatus*, *L. jensenii*, *L. iners*, *G. vaginalis*, *A. vaginae*, *Eggerthella* sp., *Leptotrichia*/*Sneathia* sp. *Megasphaera* sp., and *Prevotella* sp. (Table 1) [19]. The optimal reaction conditions and parameters were obtained by adjusting the concentration of primers, reaction temperature, and the cycles of amplification. Each qPCR contained 1 *μ*L of template DNA, 12.5 *μ*L of 2 × Takara Perfect Real Time master mix, 0.3 *μ*L of a 10 *μ*M F/R primer mix, and 10.9 *μ*L of water. The reaction parameters were as follows: 1 cycle of predenaturation for 3 min at 95°C, 40 repeated cycles of denaturation for 30 s at 94°C, annealing for 30 s, extension for 30 s at 72°C, and a final extension step for 5 min at 72°C. At the end of the qRT-PCR, the specificity of the products produced was confirmed through a melting curve analysis, whereby the PCR products were slowly heated from 55°C to 95°C in 1.0°C increments with continuous fluorescence collection.

Serially diluted standards of plasmid DNA (1 to 10^8^ copies) containing the respective amplicon for each primer pair were run on the same plate to construct a standard curve, in order to obtain a corresponding target DNA copy number of each species in each *μ*L of crude DNA template. All samples including nontemplate controls were assayed three times on separate plates. Aliquots (4 *μ*L) of the real-time PCR products with each of the species-specific primer pairs were examined by 1.5% agarose gel electrophoresis containing 0.5 mg/mL ethidium bromide.

### 2.5. Statistical Analysis

The data were expressed as the mean and standard deviation for continuous variables and as absolute and relative frequencies for categorical variables. The abundance of genus-specific bacteria was expressed as the percentage of the accurate copy numbers of the individual bacterial subgroups and total bacteria in the samples. The mean values of each item for continuous variables between two groups were compared using the Mann–Whitney *U* test, whereas differences among the three groups were analyzed using the Kruskal–Wallis *H* test. Nonparametric chi-square tests were used to compare categorical variables. Spearman's rank correlation was used to evaluate the relationship between parameters. All statistical tests were performed using SPSS, version 19.0 (SPSS Inc., Chicago, IL, USA), and any *p* value < 0.05 was considered statistically significant.

## 3. Results

### 3.1. Characteristics of the Subjects

In this study, vaginal samples of 83 healthy pregnant women were analyzed. The varying gestational ages of the women screened were compared in terms of clinical characteristics (e.g., maternal age, gravity, and parity), laboratory characteristics (vaginal pH, vaginal cleanliness, and H_2_O_2_ levels), and the presence of leukocyte esterase (Table 2). There was no difference in the maternal ages, gravidity, or parity among the three trimester groups, indicating that the participants in each of the different groups had similar clinical characteristics and were therefore comparable throughout this study. Comparing vaginal cleanliness and leukocyte esterase, it was found that they were significantly higher in women in the first trimester than in the second (*p*=0.001) and third trimester (*p* < 0.001). However, vaginal pH and H_2_O_2_ levels showed no difference among trimesters.

### 3.2. Distribution of Vaginal Bacteria in Pregnant Women

**Figure 1:**
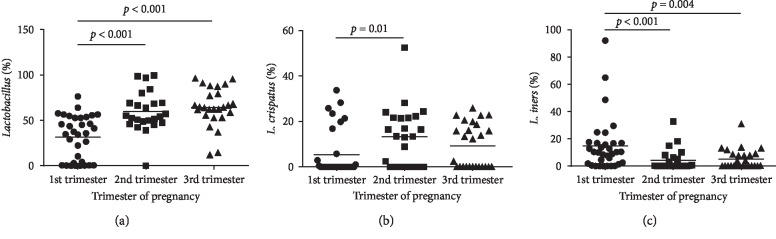
Comparison of the relative abundance of (a) *Lactobacillus*, (b) *L. crispatus*, and (c) *L. iners* in 83 asymptomatic pregnant women according to their trimester of pregnancy. Data are expressed as scatter plots, in which the horizontal lines illustrate the mean value of each genus-specific bacterium.

In this study, using qRT-PCR, seven known abundant genera (*Lactobacillus*, *Gardnerella*, *Atopobium*, *Megasphaera*, *Eggerthella*, *Leptotrichia/Sneathia*, and *Prevotella*) were analyzed. The abundance of *Gardnerella*, *Atopobium*, *Megasphaera*, *Eggerthella*, *Leptotrichia*/*Sneathia*, and *Prevotella* was significantly different among the three trimesters (*p* > 0.05). The genus *Lactobacillus* constituted the major proportion of the vaginal microbiota in healthy pregnant women, which was consistent with previous studies [34, 36, 37]. *L. jensenii*, *L. iners*, and *L. crispatus* were the most frequent species [37–39]. Among them, the abundance of *L. iners* and *L. crispatus* was significantly different among the trimesters. We also found that *L. iners* decreased significantly in women in the second and third trimester when compared with women in the first trimester (*p* < 0.001), while *L. crispatus* significantly increased in the second trimester (*p*=0.030) (Figure 1). However, the relative abundance of *L. jensenii* was unchanged obviously among the three trimesters (*p*=0.633; Table 3).

### 3.3. Correlation of Vaginal Bacteria with Vaginal Cleanliness and Leucocyte Esterase

Vaginal cleanliness and leukocyte esterase varied significantly throughout the pregnancy trimester. Spearman's rank correlation was used to examine the relationship between abundant vaginal microbiota and the two indicators including vaginal cleanliness and leukocyte esterase (Table 4). We found that vaginal cleanliness was significantly correlated with the presence of *Lactobacillus*, *Atopobium*, and *Prevotella* instead of other bacteria genus. However, all abundant bacteria except for *Megasphaera* (*p* < 0.05) were altered significantly between leukocyte esterase positive and negative. Additionally, it was interesting to find that the relative abundance of *L. iners* increased with raised vaginal cleanliness grade and positve leukocyte esterase, but *L. crispatus* did not show such similar changing patterns.

### 3.4. Relationship between Vaginal pH and Vaginal Abundant Bacteria

**Figure 2:**
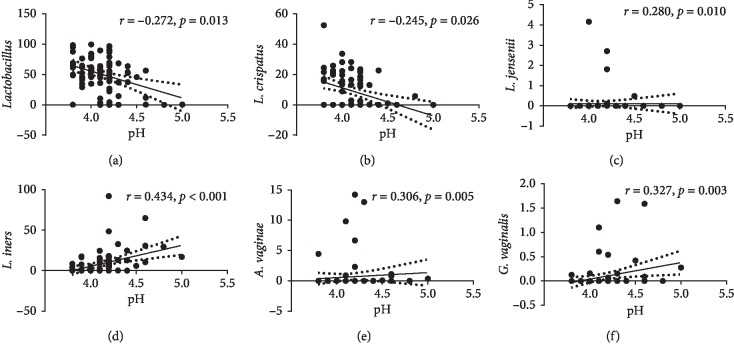
Correlation between the relative abundance of genus-specific bacteria and vaginal pH in 83 asymptomatic pregnant women. Relationships among the vaginal pH with (a) *Lactobacillus*, (b) *L. crispatus*, (c) *L. jensenii*, (d) *L. iners*, (e) *A. vaginae*, and (f) *G. vaginalis* are shown as scatter plots and regression lines. The coefficient is taken from Spearman's correlation test.

In our present study, vaginal pH was maintained at a low level in these pregnant women and was not changed significantly across the three trimesters. Normally, *Lactobacilli* acidify the vagina with lactic acid, while the overgrowth of those vaginal pathogenic bacteria may lead to the elevation of vaginal pH. We used Spearman's rank correlations to investigate the relationships between the relative abundance of genus-specific vaginal bacteria and vaginal pH. As shown in Figure 2, the vaginal pH increased when *Lactobacillus*, and more specifically *L. crispatus* decreased when the vaginal pH increased. However, the vaginal pH also was positively correlated with *L. jensenii*, *L. iners*, *A. vaginae*, and *G. vaginalis*.

## 4. Discussion

Vaginal cleanliness and leukocyte esterase have been proven previously to be associated with genital inflammation [27]. In this study, it was discovered that vaginal cleanliness and leukocyte esterase were significantly higher in women who were in the first trimester instead of the second and third trimester. Although this result cannot fully illustrate that these two inflammatory markers change spontaneously as pregnancy progressed, previous studies indicated that this phenomenon may be possible. Waters et al. performed a longitudinal investigation and found that most women who were BV positive in early pregnancy became BV negative in the third trimester [40]. Duff et al. have reported that BV disappeared spontaneously between 15 and 36 weeks in 11% of pregnant patients without any therapies or interventions [41]. However, further longitudinal studies are needed to explore the definite relationship between the dynamic vaginal microbiota and the progress of pregnancy.

Vaginal pH is another known indicator of vaginal health which is commonly used for the diagnosis of genital infections. The vaginal pH significantly increases when BV or trichomoniasis vaginitis is present and it decreases when Candida vaginitis occurs [42, 43]. However, vaginal pH did not seem to be closely associated with changes in the vagina microecosystem strongly according to the results. Furthermore, H_2_O_2_ is known as an important factor of vaginal health and is mostly produced by *Lactobacillus* and acts as a certain antibacterial compound which can protect against genital tract pathogens. Our data indicated that there was no significant difference in the presence of H_2_O_2_ among the three trimesters (*p*=0.125), indicating that it could help to maintain vaginal homeostasis in the normal status. Of course, the bacterial interactions in the pregnancy-related vaginal microbiota are complex and are not simply related to the presence or absence of H_2_O_2_-producing *Lactobacillus* [44].

The vaginal microbial community is typically characterized by abundant lactobacilli. In our study, two species of *Lactobacillus*, such as *L. iners* and *L. crispatus*, were found to be significantly different among the three trimesters. *L. iners* was found to be significantly decreased whereas *L. crispatus* was found to be significantly increased in women in the second and third trimester compared with the first trimester. Besides, we observed that vaginal cleanliness and leukocyte esterase were significantly affected by *L. iners* but not by *L. crispatus*, which was consistent with the previous study [45]. With recent advances in culture-independent community profiling, the nutritionally fastidious *L. iners* is emerging as a dominant organism, present in both healthy and *lactobacilli*-deficient aberrant vaginal environments, suggesting that *L. iners* is very flexible and can easily adapt to the fluctuating vaginal niche [46–48]. Therefore, the existence of *L. iners* may not suppress the proliferation of other potentially harmful bacteria in the vagina [49]. *L. iners* has been thought to be a precursor to being susceptible to adverse pregnant sequelae [50]. Simultaneously, *L. crispatus* has been previously suggested to be linked to healthy microbiota, a healthy pregnancy, and a term delivery and to the absence of vaginal infection or inflammation [51]. In this work, we had identified a positive correlation between inflammatory laboratory characteristics and *L. iners*.

In fact, there is a lot of controversy over whether *L. iners* is advantageous or detrimental for the host microbiota. Gajer et al. observed that the vaginal communities dominated by *L. crispatus* changed into a community dominated by *L. iners* during menstruation [52]. Their findings indicate that *L. iners* may help in the recovery of *lactobacilli-*dominant vaginal microbiota, supporting the notion that *L. iners* is a beneficial species [53]. Most women with an *L. iners*-dominated vaginal community will deliver at term with no adverse pregnancy outcome [50, 51]. Previous studies have found that *L. iners* may be a beneficial vaginal bacterium which showed beneficial effects in several mechanisms. It has been proposed that *L. iners* activates Toll-like receptor signaling in epithelial cells, elevates the levels of heat-shock protein 70, and inhibits autophagy [54, 55]. This process could combat a nonphysiological threat and maintain and promote a return to healthier conditions [56]. This would be by inducing the innate immune system in the vaginal epithelial cells, preventing harmful bacteria from obtaining important nutrients such as iron, and inhibiting their continual growth [45]. However, several studies indicated that *L. iners* offers less protection against vaginal dysbiosis [45]. In the present study, we also found that *L. iners* was more responsive to the dysbiosis of vaginal microbiota when compared to *L. crispatus*. Due to the significant complex and diverse individual discrepancies of the vaginal microbiota, more studies are required to make clear whether *L. iners* can be used as a novel biomarker to detect the presence or prognosis of vaginal inflammation and guide clinical treatment.

Our research had also explored several anaerobic microorganisms, *Gardnerella*, *Atopobium*, *Megasphaera*, *Eggerthella*, *Leptotrichia*/*Sneathia*, and *Prevotella*, which were considered as the vaginal pathogenic community. Our previous studies have found that these bacteria in the vaginal pathogenic community increased significantly in patients with BV accompanying with the depletion of *Lactobacillus* [19, 57, 58], which is highly accurate for BV diagnosis. However, the abundance of these bacteria was not changed significantly among the three trimesters in our present study. Previous studies have shown that *G. vaginalis* (belonging to Actinobacteria) plays an important role in the development of BV [19, 34]. Another genus in Actinobacteria, *Atopobium*, has been suggested to be even more specific than *G. vaginalis* for the diagnosis of BV (77% and 35%, respectively) [59]. Additionally, the presence of *Eggerthella* has also been considered as an independent risk factor for BV scores (Nugent score ≥ 7) [31]. Other members such as *Prevotella* (belonging to *Bacteroidetes*), *Megasphaera* (especially *Megasphaera type*I), and *Leptotrichia/Sneathia* (belonging to Fusobacteria) also appeared to have a strong positive association with BV, although previous research could not detect these genera in all BV samples [19, 34, 60, 61]. However, the clinical significance of these genera associated with BV in the vaginal ecosystem was still unknown. In this study, we found these bacteria in subjects who did not have BV, and therefore, this may suggest that these species may not be a specific marker for BV. Of course, these bacteria in the vaginal microbiota were interacted with each other closely. Previous study also found a clear negative association between *L. iners* and *L. gasseri* and between *A. vaginae* and *L. gasseri* [62]. Recently, Petrova et al. have showed that *L. iners* has more complex nutritional requirements and a Gram-variable morphology when compared to other *Lactobacillus* species [49]. Genome sequencing revealed that *L. iners* has an unusually small genome indicative of a parasitic or symbiotic lifestyle in the human vagina, which encodes inerolysin, a pore-forming toxin related to vaginolysin of *G. vaginalis* [49]. Similar to our previous studies, the *Lactobacillus* species, such as *L. iners* and *L. crispatus*, maintained the balance of the vaginal ecosystem, while these vaginal pathogenic bacteria contributed to the dysbiosis of the vaginal microbiota [19, 57, 58]. The depletion of *lactobacilli*, together with the increase of different species of anaerobes, could result in the switch from normal to a dysbiosis vaginal microbiota, which contributed to various adverse outcomes.

Our findings could have important implications when interpreting the varied results of investigations aimed at improving pregnancy outcomes. These data support the observation that the prevalence of vaginal microbiota varies significantly over the course of pregnancy, with a strong trend towards a reduction in infection by the third trimester. The limitation of this study is small sample capacity and nonlongitudinal design.

## 5. Conclusion

In summary, we investigated the changes of laboratory characteristics and the presence of certain bacteria in the vagina throughout pregnancy and highlighted their relationship. *L. iners* was found to be significantly decreased in the second and third trimester compared with the first trimester, while *L. crispatus* increased only in the second trimester. It was found that *L. iners* may be highly associated with vaginal dysbiosis, and further research studies are required to identify it. Additionally, quantification of the relative and absolute numbers of *L. iners* under different conditions throughout pregnancy was needed to be able to potentially predict adverse outcomes that it may be associated with. This study was limited due to the relatively small sample size (*n* = 83); however, it did provide the basis of future work to investigate the role of the microbiota in both low- and high-risk pregnancies.

## Figures and Tables

**Table 1:** Species-specific primer sets for detection of vaginal bacteria by quantitative real-time PCR.

PCR specificity	Primer	Sequence (5′-3′)	Annealing temp.	Amplicon size (bp)	Reference
All bacteria	Bac27F	AGAGTTTGATCCTGGCTCAG	65°C	312	[28]
	EUB338R-I	GCTGCCTCCCGTAGGAGT			

*Lactobacillus* genus	Bact-0011	AGAGTTTGATYMTGGCTCAG	62°C	667	[29]
	Lab-0677	CACCGCTACACATGGAG			

*L. crispatus*	Lcris-F	AGCGAGCGGAACTAACAGATTTAC	65°C	154	[30]
	Lcris-R	AGCTGATCATGCGATCTGCTT			

*L. jensenii*	Ljens-F	AAGTCGAGCGAGCTTGCCTATAGA	60°C	162	[31]
	Ljens-R	CTTCTTTCATGCGAAAGTAGC			

*L. iners*	Liners-F	CTCTGCCTTGAAGATCGGAGTGC	65°C	155	[31]
	Liners-R	ACAGTTGATAGGCATCATCTG			

*Atopobium vaginae*	AV-F	TAGGTCAGGAGTTAAATCTG	62°C	156	[32]
	AV-R	TCATGGCCCAGAAGACCGCC			

*Gardnerella vaginalis*	GV1-F	TTACTGGTGTATCACTGTAAGG	62°C	332	[33]
	GV3-R	CCGTCACAGGCTGAACAGT			

*Eggerthella*	Egger-621F	AACCTCGAGCCGGGTTCC	60°C	239	[34]
	Egger-859R	TCGGCACGGAAGATGTAATCT			

*Leptotrichia/Sneathia*	Lepto-395F	CAATTCTGTGTGTGTGAAGAAG	60°C	252	[34]
	Lepto-646R	ACAGTTTTGTAGGCAAGCCTAT			

*Megasphaera type I*	MegaE-456F	GATGCCAACAGTATCCGTCCG	64°C	212	[34]
	MegaE-667R	CCTCTCCGACACTCAAGTTCGA			

*Prevotella*	Prevo-F	CCAGCCAAGTAGCGTGCA	60°C	151	[35]
	Prevo-R	TGGACCTTCCGTATTACCGC			

**Table 2:** Clinical and laboratory characteristics of the participants.

Parameters	Participants in 1st trimester (*n* = 33)	Participants in 2nd trimester (*n* = 24)	Participants in 3rd trimester (*n* = 26)	*p* value
Age (years)	28.30 (5.35)	29.50 (3.91)	29.35 (4.03)	0.221
Gravidity	0.88 (0.96)	1.21 (1.22)	1.31 (1.62)	0.604
Parity	0.55 (0.66)	0.50 (0.51)	0.42 (0.50)	0.832
PH	4.2 (0.3)	4.0 (0.2)	4.1 (0.2)	0.118
Cleanliness (1–2/3–4)	18/15	23/1	22/4	**0.001**
H_2_O_2_ (<2/≥2)	5/28	0/24	2/24	0.125
Leucocyte esterase (P/N)	14/19	0/24	4/22	**<0.001**

All data are mean (standard deviation). P, positive; N, negative.

**Table 3:** Comparison of the relative abundance of vaginal bacteria by quantitative real-time PCR according to trimesters of pregnancy.

Species	Participants in 1st trimester (*n* = 33)	Participants in 2nd trimester (*n* = 24)	Participants in 3rd trimester (*n* = 26)	*p* value
*Lactobacillus genus* (%)	31.57 (23.94)	59.80 (21.96) b	64.14 (21.61) b	<0.001
*L. jensenii* (%)^a^	0.07 (0.32)	0.43 (2.07)	0.27 (1.31)	0.633
*L. crispatus* (%)^a^	5.74 (10.39)	13.21 (12.96) c	9.67 (10.47)	0.030
*L. iners* (%)^a^	14.86 (19.74)	4.16 (7.97) d	5.02 (7.24) d	<0.001
*Atopobium vaginae* (%)	1.21 (3.17)	2.63 (12.86)	0.03 (0.16)	0.897
*Gardnerella vaginalis* (%)	0.10 (0.24)	0.08 (0.34)	2.49 (12.67)	0.944
*Eggerthella* (%)	0.05 (0.21)	0.00 (0.00)	0.00 (0.00)	0.466
*Leptotrichia*/*Sneathia* (%)	0.04 (0.12)	0.00 (0.00)	0.00 (0.00)	0.432
*Megasphaera type* (%)	0.29 (0.80)	0.02 (0.10)	0.02 (0.09)	0.569
*Prevotella* (%)	0.19 (0.45)	0.00 (0.01)	0.09 (0.45)	0.169

All data are mean (standard deviation). ^a^The relative abundance of *L. crispatus*, *L. jensenii*, and *L. iners* was compared to the copy number of *Lactobacillus* genus; ^b^compared with 1st trimester, *p* < 0.001; ^c^compared with 1st trimester, *p* < 0.05; ^d^compared with 1st trimester, *p* < 0.01.

**Table 4:** Correlation of the relative abundance of vaginal bacteria with vaginal cleanliness and leucocyte esterase.

Vaginal bacteria	Vaginal cleanliness			Leucocyte esterase		
	1–2 (*n* = 63)	3–4 (*n* = 20)	*p* value	Negative (*n* = 65)	Positive (*n* = 18)	*p* value
*Lactobacillus* genus (%)	55.00 (25.14)	34.10 (27.18)	0.002	55.42 (23.86)	30.13 (29.06)	0.001
*L. jensenii* (%)	10.68 (11.91)	4.27 (8.67)	0.072	10.39 (11.84)	4.61 (9.09)	0.241
*L. crispatus* (%)	0.30 (1.53)	0.02 (0.22)	0.873	0.29 (1.51)	0.03 (0.47)	0.220
*L. iners* (%)	6.18 (13.04)	16.56 (16.49)	**<0.001**	6.18 (12.79)	17.73 (17.15)	**<0.001**
*Atopobium vaginae* (%)	0.51 (2.46)	1.09 (2.57)	0.044	0.49 (2.42)	1.23 (2.67)	0.004
*Gardnerella vaginalis* (%)	0.05 (0.22)	0.20 (0.43)	0.133	0.04 (0.22)	0.23 (0.44)	0.023
*Eggerthella* (%)	0.00 (0.01)	0.08 (0.84)	0.194	0.00 (0.01)	0.09 (1.02)	0.006
*Leptotrichia/Sneathia* (%)	0.01 (0.11)	0.04 (0.21)	0.496	0.00 (0.01)	0.06 (0.41)	0.032
*Megasphaera type* (%)	0.07 (0.23)	0.30 (0.75)	0.686	0.06 (0.43)	0.36 (1.32)	0.097
*Prevotella* (%)	0.02 (0.18)	0.36 (1.46)	0.004	0.01 (0.02)	0.45 (1.36)	0.002

All data are mean (standard deviation). The abundance of vaginal bacteria relative to total bacteria gene copy number according to vaginal cleanliness and leucocyte esterase was compared. ^a^The relative abundance of *L. crispatus*, *L. jensenii*, and *L. iners* was compared to the copy number of *Lactobacillus* genus.

## Data Availability

The data used to support the findings of this study are available from the corresponding author upon request.

## Supplementary Materials

Supplementary Materials Table S1: judgment method of vaginal cleanliness.
