# Supplementary material for: Lactobacillus iners Is Associated with Vaginal Dysbiosis in Healthy Pregnant Women: A Preliminary Study
Source: Biomed Res Int. 2019 Oct 23;2019:6079734. doi: 10.1155/2019/6079734 (PMC6855029; doi:10.1155/2019/6079734)
Supplement: Supplementary Materials — Table S1: judgment method of vaginal cleanliness. [file 6079734.f1.docx]

**Table S1 Judgment method of vaginal cleanliness**

| Vaginal cleanliness | Lactobacillus | Epithelial cells | White blood cells | Other flora |
| --- | --- | --- | --- | --- |
| Degree Ⅰ | 30/OML | Full field | 0-5/HPL | None |
| Degree Ⅱ | 6-30/OML | 1/2 field | 5-15/HPL | 1-5/OML |
| Degree Ⅲ | 1-5/OML | Less than 1/2 field | 15-30/HPL | 6-3/OML |
| Degree Ⅳ | <1/OML | None | >30/HPL | >30/OML |

OML: Oil immersion lens; HPL: high- power lens
